# Reimagining the journey to recovery: The COVID-19 pandemic and global mental health

**DOI:** 10.1371/journal.pmed.1004224

**Published:** 2023-04-24

**Authors:** Vikram Patel, Daisy Fancourt, Toshi A. Furukawa, Lola Kola

**Affiliations:** 1 Department of Global Health and Social Medicine, Harvard Medical School, Boston, Massachusetts, United States of America; 2 Institute of Epidemiology and Health, University College London, London, United Kingdom; 3 Department of Health Promotion and Human Behavior, Kyoto University Graduate School of Medicine, Kyoto, Japan; 4 World Health Organization Collaborating Center for Research and Training in Mental Health, Neuroscience and Substance Abuse, Department of Psychiatry, College of Medicine, University of Ibadan, Ibadan, Nigeria

## Abstract

In this editorial, guest editors Vikram Patel, Daisy Fancourt, Lola Kola, and Toshi Furukawa discuss the contents of the special issue on the pandemic and global mental health, highlighting key themes and providing important context.

On March 11, 2020, the World Health Organization (WHO) declared the Coronavirus Disease 2019 (COVID-19) outbreak a global pandemic [1]. The world has since been transformed by the Severe Acute Respiratory Syndrome Coronavirus 2 (SARS-CoV-2) virus, with lockdowns and physical distancing decimating social support structures. Loss of income, school closures, disruptions in routine health care, and reductions in physical activity have widened preexisting health inequities [2]. We have learned much about this novel virus and miraculous new ways of preventing its worst outcomes. The pandemic has also reminded us that social connectedness and solidarity are essential, with disruptions of our social behaviors having manifold implications for individual and societal well-being. We have also been reminded of the need for clear and transparent communication of science and the necessity of universal health coverage, stewarded by the state, with a strong population health and equity focus. And, of course, the pandemic has highlighted the critical importance of mental health to overall well-being. For those of us who have often complained of the low priority afforded to mental health, surely this is a truly historic moment that we must not let slip from our grasp.

While much has been written about the mental health crisis in the shadow of the pandemic, let’s be clear of one fact: The world was experiencing a mental health crisis even before the pandemic. The mental health crisis is reflected in many ways, such as the unchanging and, in some contexts and populations, the rising prevalence of mental illness, substance use problems, and self-harm. Prior to the pandemic, suicide was the second leading cause of death in young people globally, opioid overdose had become a leading cause of death in middle-aged Americans, and 76% to 85% of people with mental health conditions in low- and middle-income countries (LMICs) received no care from the health system for their condition [3]. The exacerbation of social adversities and inequities recognized as risk factors for poor mental health, such as poverty and gender-based violence, the prolonged periods of uncertainty, the restrictions on fundamental social behaviors, the experience of virus-related sickness and loss of loved ones, and the disruption of essential developmental tasks which are central to youth mental health, were feared as adding fuel to the mental health problem burden in all countries. These fears were further confirmed by a modeling analysis published 2 years into the pandemic [4].

Yet, as we enter the fourth year of the pandemic, it appears that the worst is now behind us, with a range of factors contributing to dramatically reduced risks of mortality associated with the virus. Most countries appear to be returning to a pre-pandemic cadence of life and, understandably, many of us seek to simply move on from the pandemic. However, it is important to derive lessons on its impact on health and social circumstances. A “postmortem” is necessary to understand the consequences of policies, often implemented at a time when there was very poor knowledge about the virus itself, but to also prepare societies for future pandemics that are, in the context of ever greater conflict between human settlements and the natural world, increasingly likely. Further, the landscape remains volatile, with new variants of variable infectivity and lethality still emerging, and remaining uncertainty over inequitable access to vaccines and antivirals [5,6].

Moreover, mental health problems are often known to run chronic courses and could have enduring consequences. We are yet to witness the long-term impact of disrupted lives, especially for the younger generations, or the effects that socioeconomic adversities at least partly attributable to the pandemic (such as recessions, increased social inequalities, and civil unrest) will have over the coming years. This Special Issue of *PLOS Medicine* set out to document research examining mental health aspects of the pandemic with the potential to mitigate such mental health consequences, strengthening the global response to future pandemics, while informing mental health policy and practice more generally as societies are exhorted to “build back better” [7]. We were particularly interested in research which addressed vulnerable populations and the pandemic’s impact on existing mental health inequities; health system responses to increased demand for mental health care; evaluations of policy interventions which may have had positive or harmful effects on mental health; and mental health consequences from a life-course perspective. The articles in this Issue cover most of these themes and offer important insights into the questions which are of interest to the field.

First, these papers demonstrate that the increases in prevalence of mental health problems expectedly were primarily concentrated in mood and anxiety conditions, but not severe mental health problems [8–13], and, some conditions even showed a decline or inconclusive findings (for example, self-harm was associated with self-reported symptoms but not serology confirmed infection in one study) [14]. Importantly, while one systematic review reported that symptoms of mood and anxiety problems increased in the first 6 months after the onset of the pandemic, the picture is less clear when participants are followed over longer periods and some changes may have been transient [15–17]. Second, there was strong association of the prevalence of mental health problems with contextual factors such as the stringency of lockdowns [9] and severity of the pandemic [8,9,11] and prevailing socioeconomic factors (such as agricultural production) [12]. Weaker associations were observed for a range of other subjective factors, such as confidence in the government or health care, COVID-19 knowledge, personal COVID-19 infection, and social support [9,18]. Notably, the strength of some of these associations fluctuated during the first 2 years of the pandemic, with certain factors, such as higher national death rates, individual fears about catching COVID-19, and worries about accessing essentials like food and water, weakening as predictors of poorer mental health over time. This highlights that, while we can anticipate certain factors that will be triggers for worsening prevalence of mental illness, we have to remain alert during pandemics to their constantly changing nature and the extraordinary circumstances they give rise to in designing responsive context-specific public policies [9]. Reassuringly, there seemed to be no sustained ill-effects of working from home, at least in a specific high-income context, though there might be subgroup variations [19]. Third, the increased burden of common mental health problems was inequitably distributed, particularly affecting women, young people, and low-income groups, exposing and widening fault lines that exist in the foundations of our societies [8,10,11,18]. The increased use of mental health services in specific occupational groups, notably health-care workers, has also been demonstrated [20], though a decline in mental health in health-care workers during the pandemic was not a consistent observation [18]. More worryingly, there was a clear demonstration of discrimination in terms of access to COVID life-saving interventions for people with severe mental health problems, which was possibly a key factor contributing to their higher mortality [21].

In summary, these papers show that the impact of the pandemic on mental health has been selective (with a particular impact on mood and anxiety problems), influenced by time-varying contextual factors related to the pandemic and consistent with social determinants that were well recognized before the pandemic. This body of evidence is both consistent with other reviews on this subject [4,15,17,22] but also suggests that, contrary to our worst fears, populations globally may have navigated these extraordinary years of uncertainty and loss with remarkable resilience and fortitude. That said, there might be important contextual differences in these observations. Thus, among the handful of publications with data from LMIC, one reported the remarkable finding that QALYs lost due to morbidities (much of this related to mental health) was 5 to 11 times greater than those lost due to COVID-19 premature mortality [11], while another study observed that the negative effect of the pandemic on mental health was of similar magnitude to the positive effect of multifaceted anti-poverty programs [12]. Further, the contexts in which much of the research was conducted even as recently as 2022 have witnessed dramatic changes as they deal with the fallout of the pandemic (for example, a return to in-person work and travel) and the global economic uncertainties due to a multitude of factors, including the war in Ukraine.

Future research actions need to address limitations which are evident in the studies documented in this Special Issue and true of most mental health research: limited generalizability (in particular, the paucity of research in the global health context), the reliance on self-reported outcomes, high attrition and non-participation rates (no doubt, unique to the circumstances of conducting research during the pandemic), and heterogenous approaches to measuring mental health. These limitations make a strong case for standardizing outcome measures, deployment of digital technologies for real-time assessments and electronic health data collection where feasible, designing and evaluating novel interventions which target risk factors for poor mental health and mental health disparities, and better characterization of predictors of treatment response (the holy grail of precision medicine in psychiatry).

This Special Issue and other developments during the pandemic offer a prism through which we can examine how the mental health landscape may change in the coming years, reassessing our policy and practice priorities. The public health emergency posed by COVID-19 required coordinated joint efforts from national governments, global health communities and all mental health stakeholders in the public and private sectors [23]. The United Nations and WHO have committed to increase mental health and psychosocial support (MHPSS) in their COVID-19 response efforts across sectors [24]. The pandemic has witnessed a dramatic increase in awareness and concern about mental health and fueled rapid adoption of novel digital technologies for care, notably telemedicine and telecare platforms as well as self-care apps. This renewed attention, solidarity, innovation, and science offers a historic opportunity to reimagine mental health. That said, there remain large unmet needs for care that, along with the global economic uncertainties, are fueling poor mental health globally. Placing this Special Issue in the broader context of interdisciplinary mental health science, notably the large body of epidemiological science on the social determinants of mental illness, clinical science on the opportunities for early intervention and the efficacy of psychosocial interventions, and delivery science on the effectiveness of the delivery of these interventions by front-line workers, lays the foundation for a transformed vision of mental health care [3].

Ultimately, this body of science sends a message of hope: Most individuals have remarkable wells of resilience to draw upon even in unprecedented times that are unimaginably difficult, but these are heavily influenced by inequities, social determinants, actions by the state, and compassion by others in their communities. This evidence facilitates a move from a nihilistic view about the lack of evidence to a hopeful view, where a suite of evidence-based interventions, spanning policy actions at the societal level to individual actions in personal encounters, can be marshaled for the prevention and care of mental health problems. That is the heart of a reimagined journey to recovery, both for our societies that face immense challenges and for the individuals who experience mental health problems.
